# Perception of People Diagnosed With Fibromyalgia About Information and Communication Technologies for Chronic Pain Management: Cross-Sectional Survey Study

**DOI:** 10.2196/55751

**Published:** 2024-06-18

**Authors:** Xènia Porta, Rubén Nieto, Mayte Serrat, Pierre Bourdin Kreitz

**Affiliations:** 1 eHealthLab Faculty of Psychology and Education Universitat Oberta de Catalunya Barcelona Spain; 2 Unitat d’Expertesa en Síndromes de Sensibilització Central Servei de Reumatologia Hospital Universitari Vall d’Hebron Barcelona Spain; 3 Escola Universitària de Fisioteràpia Escoles Universitàries Gimbernat Universitat Autònoma de Barcelona Barcelona Spain; 4 Computer Sciences, Multimedia and Telecommunication Department Universitat Oberta de Catalunya Barcelona Spain; 5 XR-Lab Research Hub Universitat Oberta de Catalunya Barcelona Spain

**Keywords:** fibromyalgia, chronic pain, pain management, information and communication technologies, use, satisfaction

## Abstract

**Background:**

Chronic pain is prevalent in our society, with conditions such as fibromyalgia being notably widespread. The gold standard for aiding individuals dealing with chronic pain involves interdisciplinary approaches rooted in a biopsychosocial perspective. Regrettably, access to such care is difficult for most of the people in need. Information and communication technologies (ICTs) have been used as a way of overcoming access barriers (among other advantages).

**Objective:**

This study addressed the little explored area of how individuals with fibromyalgia use and perceive different types of ICTs for pain management.

**Methods:**

A cross-sectional study was conducted using an online survey. This survey was created to assess the use of different ICT tools for pain management, satisfaction with the tools used, and perceived advantages and disadvantages. In addition, data collection encompassed sociodemographic variables and pain-related variables, pain intensity, the impact of pain on daily life activities, and fear of movement/injury beliefs. In total, 265 individuals diagnosed with fibromyalgia completed the survey.

**Results:**

Only 2 (0.75%) participants reported not having used any ICT tool for pain management. Among those who used ICT tools, an average of 10.94 (SD 4.48) of 14 different tools were used, with the most used options being instant messaging apps, websites dedicated to managing fibromyalgia, phone calls with health professionals, and online multimedia resources. Satisfaction rates were relatively modest (mean 2.09, SD 0.38) on a scale from 0 to 5, with instant messaging apps, phone calls with health professionals, fibromyalgia management websites, and online multimedia resources being the ones with higher satisfaction. Participants appreciated the ability to receive treatment from home, access to specialized treatment, and using ICTs as a supplement to in-person interventions. However, they also highlighted drawbacks, such as a lack of close contact with health professionals, difficulty expressing emotions, and a lack of knowledge or resources to use ICTs. The use of ICTs was influenced by age and educational background. Additionally, there was a negative correlation between satisfaction with ICT tools and fear of movement/injury.

**Conclusions:**

People with fibromyalgia are prone to using ICTs for pain management, especially those tools that allow them to be in contact with health professionals and have access to online resources. However, there is still a need to improve ICT tools since satisfaction ratings are modest. Moreover, strategies aimed at older people, those with lower levels of education, and those with higher levels of fear of movement/injury can be useful to potentiate the use of ICTs among them.

## Introduction

Chronic pain is a prevalent issue in our society. The number of cases leads to an enormous cost at the societal level and causes suffering for both people with chronic pain and their relatives [1,2]. To help people with chronic pain, multidisciplinary interventions, viewed through a biopsychosocial lens, stand as the gold standard for addressing chronic pain. Unfortunately, access to these interventions is often challenging due to the limited availability of multidisciplinary units and insufficient training in pain management among health care professionals [3-5]. Information and communication technologies (ICTs) represent an opportunity for facilitating access to evidence-based interventions at an affordable cost. Furthermore, ICTs can increase autonomy and empower individuals to become more actively involved in their own care [6]. For these reasons, their use is increasing in the health field in general [7,8] but also for people with chronic pain [9,10]. The COVID-19 pandemic has increased the awareness and potential of ICTs [11].

Despite the considerable potential, little research has explored to what extent people with pain use ICTs and what their perceptions about ICTs are. Understanding how they use available solutions and what their impressions are can help in advancing the field by designing better solutions or improving the access to the ones available that are not used by people, in addition to showing them to be effective.

Along these lines, Ledel et al [12] conducted a qualitative study to examine the experiences of patients with chronic pain with regard to ICTs and identify possible facilitators of and barriers to patients’ use of eHealth for pain management. The patients were, in general, in favor of using ICTs for pain (apps were the preferred tool). They also emphasized the necessity of having access to technological tools under any circumstance as a facilitator of using ICTs in general, as well as being able to use them appropriately regardless of their level of pain or ability to concentrate.

Schneider and Hadjistavropoulos [13] conducted a survey to investigate the impressions of 129 participants with chronic pain about internet-based cognitive behavioral therapy (ICBT) and to identify factors that are associated with a willingness to consider ICBT. Participants generally agreed that ICBT is beneficial, especially for patients in rural areas, those with mobility issues, and those who have difficulty attending appointments.

Cranen et al [14] explored chronic pain patients’ perspectives on potential telerehabilitation services through semistructured interviews of 25 participants. In general terms, they found that factors influencing patients’ perceptions about telerehabilitation are complex and different among users. For example, patients saw benefits in telerehabilitation but were hesitant to consider it as a stand-alone treatment due to concerns about performance expectancy. The preference for face-to-face interaction with therapists was highlighted as crucial for receiving effective feedback and exercise guidance, particularly during the initial stages of treatment. In a subsequent study, in which they explored various treatment characteristics, Cranen et al [15] discovered that the most favored treatment approach is an “intermediate” scenario. This scenario combines conventional clinic-based rehabilitation and a telerehabilitation program focused on self-management. The study underscores the potential of remote feedback and monitoring technology in chronic pain telerehabilitation and highlights the need for patient-centered treatment design.

In addition to these preliminary findings, further research is needed to investigate the use of and satisfaction with a wide variety of ICTs for pain treatment or management. Much of the existing literature is limited to specific ICT solutions, and larger-sample studies focusing on specific pain problems are warranted, as needs and impressions may vary among different groups [16]. Therefore, this study aimed to fill this gap by comprehensively exploring the potential use of and satisfaction with a broad range of ICTs for the management of pain in individuals with fibromyalgia.

Fibromyalgia is 1 of the most prevalent conditions contributing to recurring pain [17]. It is a chronic and complex condition that causes widespread pain and profound exhaustion, as well as a variety of other accompanying symptoms, such as fatigue, stiffness, and sleep disturbances. Moreover, individuals with fibromyalgia often experience altered pain perception and processing, making them more sensitive to pain compared to the general population. The prevalence of fibromyalgia worldwide is between 0.2% and 6.6% [18], with a greater rate in women, whose prevalence values are placed around 3.4%, while for men, the prevalence is around 0.5% [19]. These numbers, and the specificity of this condition, merit a study focused on them.

More specifically, we wanted to provide evidence about the ICT tools most frequently used by people with fibromyalgia, the most valuable tools for them, and the most important advantages and disadvantages. We also wanted to test patients’ use and perceptions of ICT tools vary, considering sociodemographic and pain-related variables. This is because we hypothesized that although people with fibromyalgia have similar health problems, their use and perceptions of different ICT tools can vary depending on their specific situation.

## Methods

### Procedure

The study was conducted between December 1 and 14, 2022. An online survey was designed to gather cross-sectional data. Completing the survey required approximately 30 minutes.

### Ethical Considerations

The study was approved by the Ethics Committee of the Universitat Oberta de Catalunya (UOC). Participants were asked to provide signed informed consent within the online survey. Their participation was completely voluntary, without any reward, and they remained anonymous.

### Study Population

Participants were recruited by disseminating the study information to lists of individuals who self-reported having been diagnosed with fibromyalgia and had attended a multidisciplinary intervention for pain management or were awaiting such treatment in Barcelona (Spain). In total, 265 participants who confirmed that they were diagnosed with fibromyalgia provided signed informed consent and completed the survey.

### Data Collection

A specific survey was created to achieve study objectives. Moreover, sociodemographic and pain-related variables (intensity of pain, impact/interference in activities of daily life, and fear of movement/injury beliefs) were assessed to study correlations and relations with the use of ICTs.

#### Survey

The survey was designed considering the study objectives in order to evaluate sociodemographic variables, fibromyalgia characteristics (time since the onset of symptoms/diagnosis), and, especially, uses and perceptions of ICTs for fibromyalgia management. More specifically, participants were asked about:

- ICT tools used for pain management and their associated satisfaction: A list of 14 potential types of ICTs was presented, and participants were asked to rate each on a scale from 0 (not used) to 5. The 14 types of ICTs were instant messaging apps, fibromyalgia management websites, phone calls with health professionals, online multimedia resources, social networks, video conferences with health professionals, technologies for tracking activity, digital sensors, email, mobile phone apps, cameras to assess posture and movement, platforms for following symptoms or schedules, virtual reality, and video games. We attempted to create an exhaustive list of all those technological tools that could be potentially useful for the management of pain and fibromyalgia.
- Advantages and disadvantages of the use of ICT tools: Participants were provided with a predefined list of advantages and disadvantages, from which they could select all the ones they considered appropriate. Additionally, they could provide new advantages/disadvantages not included in the list presented. Advantages included the possibility of receiving treatment from home, access to specialized treatment, use as a complement to face-to-face interventions, reduction in economic cost, and anonymity. Disadvantages included not having close contact with health professionals, not being able to express emotions and feelings, a lack of knowledge or resources to use ICTs, difficulty for the health professional to understand the patient’s nonverbal language, difficulty for the patient to understand the health professional’s nonverbal language, a lack of data confidentiality, a lack of evidence about the use of ICTs for fibromyalgia management, network band or connection problems, and a lack of legal regulations. These were defined by considering the study by Sora et al [20]. The survey was designed in the Spanish language and implemented online using the Google Forms.

#### Chronic Pain Grade Questionnaire

The Chronic Pain Grade Scale (CPGS) is a 7-item self-administered questionnaire that was developed by Von Korff et al [21] in 1992. It is designed to measure 2 important factors related to chronic pain: the level of pain intensity (characteristic pain intensity) and the degree of pain-related disability (disability score). The scores of both subscales are combined to obtain the degree of chronic pain, which is divided into 4 different categories and can range from grade I (minimal pain and disability) to grade IV (high degree of pain and highly limiting disability). The Spanish version, which has shown appropriated psychometric properties, was used for the study [22].

#### Tampa Scale of Kinesiophobia

The 11-item Tampa Scale for Kinesiophobia (TSK-11) is a self-administered questionnaire that was developed by Woby et al [23] in 2005. The TSK-11 is commonly used to assess the degree of fear of movement/injury, with higher scores being indicative of greater fear. The Spanish version, which has been validated with 2 different pain samples (a heterogeneous chronic pain sample and a musculoskeletal acute pain sample) and demonstrated to have good psychometric properties [24], was used in this study.

#### Fibromyalgia Impact Questionnaire-Revised

The Fibromyalgia Impact Questionnaire-Revised (FIQ-R) is a revised version of the Fibromyalgia Impact Questionnaire (FIQ), a fibromyalgia assessment instrument commonly used to analyze the impact of this condition on patients’ activities of daily living, updated by Bennett et al [25] in 2009. The FIQ-R consists of a total of 21 items, which are divided into 3 subscales of function, overall impact, and symptoms. A higher score indicates a greater impact of fibromyalgia on the patient’s activities of daily living. The Spanish version, which has shown good internal consistency and proven validity in adequately evaluating patients with fibromyalgia [26], was used in this study.

### Data Analysis

All data analyses were performed using SPSS Statistics version 25 (IBM Corporation). Descriptive statistics were computed for sociodemographic variables, pain-related variables, ICT tools used for pain management, satisfaction, and advantages and disadvantages. Next, bivariate correlations were computed to test potential relationships between study variables. These included the number of ICT tools used, the number of advantages/disadvantages of ICTs, the degree of satisfaction with each ICT tool listed, age, education level, time elapsed since diagnosis, and the results of the TSK-11, the FIQ-R, and the CPGS. Paired *t* tests were conducted to study differences in the number of ICT tools used and the number of advantages/disadvantages by some sociodemographic variables (ie, gender, living status [alone or accompanied], being in charge of relatives, and employment status).

## Results

### Sociodemographic and Diagnosis Variables

The study sample comprised predominantly women, constituting 244 (92.1%) of the 265 participants, with a mean age 53.81 (SD 8.44) years. In addition, 189 (71.7%) participants had completed at least secondary school, and 117 (44.1%) were either unemployed or disabled for work. Finally, 224 (84.5%) participants resided with someone else, and nearly half were in charge of dependents.

Regarding fibromyalgia, 222 (83.8%) participants had symptoms of fibromyalgia for more than 5 years, and 166 (62.6%) were diagnosed more than 5 years ago (see Table 1).

**Table 1:** Sociodemographic and diagnosis variables.

Variables		Participants (N=265), n (%)
**Education**		
	No education	2 (0.8)
	Primary education	73 (27.5)
	Secondary education	136 (51.3)
	University education	39 (14.7)
	Postgraduate education	15 (5.7)
**Employment status**		
	Employed	109 (41.1)
	Unemployed	35 (13.2)
	Retired	30 (11.3)
	Disabled for work	82 (30.9)
	Student	2 (0.8)
	Homemaker	3 (1.1)
	Others	4 (1.5)
**Living status**		
	Living alone	41 (15.5)
	Living accompanied by someone	224 (84.5)
**In charge of relatives (children); n=108, (40.8%)**		
	1	50 (46.3)
	2	51 (47.2)
	3	7 (6.5)
**In charge of relatives (adults); n=59 (22.3%)**		
	1	35 (59.3)
	2	18 (30.5)
	3	5 (8.5)
	4	1 (1.7)
Not in charge of any relatives		138 (52.1)
**Time since first symptoms**		
	<6 months	0
	Between 6 months and 1 year	1 (0.4)
	Between 1 and 2 years	9 (3.4)
	Between 2 and 5 years	33 (12.5)
	>5 years	222 (83.8)
**Time since diagnosis**		
	<6 months	1 (0.4)
	Between 6 months and 1 year	4 (1.5)
	Between 1 and 2 years	34 (12.8)
	Between 2 and 5 years	60 (22.6)
	>5 years	166 (62.6)

### Pain-Related Variables

Mean scores in the CPGS (characteristic pain intensity, pain-related disability), the TSK-11 (fear of movement/injury), and the FIQ-R (disability) were high. Considering grades computed by the CPGS, participants were classified into grade I (n=7, 2.5%), grade II (n=28, 10.6%), grade III (n=43, 16.1%), and grade IV (n=188, 70.8%). See Table 2 for details.

**Table 2:** Mean scores on pain-related variables.

Variables		Score, mean (SD); min-max
**Pain severity (CPGQ^a^)**		
	Characteristic pain intensity	76.31 (12.90); 23.33-100.00
	Pain-related disability	74.83 (16.71); 10.00-100.00
Fear of movement/injury (TSK-11^b^)		23.43 (8.11); 11.00-4.004
Disability (FIQ-R^c^)		67.85 (18.95); 10.67-100.00

^a^CPGS: Chronic Pain Grade Scale.

^b^TSK-11: 11-item Tampa Scale for Kinesiophobia.

^c^FIQ-R: Fibromyalgia Impact Questionnaire-Revised.

### ICT Tool Use and Satisfaction

Participants reported having used a mean of 10.94 (SD 4.48) tools from a total of 14 tools. Of the 265 participants, 2 (0.75%) reported not having used any of the ICT tools listed (see Table 3). The most commonly used ICT tools were instant messaging apps (n=237, 89.4%), fibromyalgia management websites (n=234, 88.3%), phone calls with health professionals (n=228, 86%), and online multimedia resources (n=222, 83.8%), while the less frequently used ones were cameras to assess posture and movement progress (n=192, 72.5%), platforms for following symptoms or schedules (n=189, 71.3%), virtual reality (n=186, 70.2%), and video games (n=186, 70.2%).

The mean degree of satisfaction for all 14 ICT tools used was 2.09 (SD 0.38). Tools that received the highest satisfaction ratings were instant messaging apps (mean 2.69, SD 1.41), phone calls with health professionals (mean 2.58, SD 1.45), fibromyalgia management websites (mean 2.53, SD 1.30), and online multimedia resources (mean 2.40, SD 1.31), while the lowest satisfaction ratings were attributed to video games (mean 1.55, SD 1.08), virtual reality (mean 1.67, SD 1.12), cameras to assess posture and movement (mean 1.71, SD 1.12), and digital sensors (mean 1.78, SD 1.23). Specific results for all the ICT alternatives can be seen in Table 3.

**Table 3:** Percentage of participants using each of the ICT^a^ tools and satisfaction level with them.

ICT tool	Participants (N=265) using the tool, n (%)	Mean (SD)	Satisfaction rating, n (%)					
			Never used	1	2	3	4	5
Instant messaging apps	237 (89.4)	2.69 (1.41)	28 (10.6)	62 (23.4)	60 (22.6)	43 (16.2)	34 (12.8)	38 (14.3)
Fibromyalgia management websites	234 (88.3)	2.53 (1.30)	31 (11.7)	64 (24.2)	64 (24.2)	47 (17.7)	36 (13.6)	23 (8.7)
Phone calls with health professionals	228 (86.0)	2.58 (1.45)	37 (14.0)	70 (26.4)	58 (21.9)	35 (13.2)	27 (10.2)	38 (14.3)
Online multimedia resources	222 (83.8)	2.40 (1.31)	43 (16.2)	71 (26.8)	60 (22.6)	44 (16.6)	25 (9.4)	22 (8.3)
Social networks	211 (79.6)	2.16 (1.31)	54 (20.4)	89 (33.6)	56 (21.1)	29 (10.9)	17 (6.4)	20 (7.5)
Video conferences with health professionals	211 (79.6)	2.35 (1.45)	54 (20.4)	88 (33.2)	42 (15.8)	29 (10.9)	24 (9.1)	28 (10.6)
Technologies for tracking activity	204 (77.0	1.83 (1.28)	61 (23.0)	127 (47.9)	30 (11.3)	16 (6.0)	17 (6.4)	14 (5.3)
Digital sensors	203 (76.6%)	1.78 (1.23)	62 (23.4)	127 (47.9)	32 (12.1)	19 (7.2)	11 (4.2)	14 (5.3)
Email	203 (76.6)	2.22 (1.31)	62 (23.4)	80 (30.2)	55 (20.8)	30 (11.3)	19 (7.2)	19 (7.2)
Mobile phone apps	193 (72.8)	1.95 (1.24)	72 (27.2)	97 (36.6)	49 (18.5)	20 (7.5)	13 (4.9)	14 (5.3)
Cameras to assess posture and movement	192 (72.5)	1.71 (1.12)	72 (27.5)	120 (45.3)	35 (13.2)	17 (6.4)	12 (4.5)	8 (3.0)
Platforms for following symptoms or schedules	189 (71.3)	1.84 (1.21)	76 (28.7)	106 (40.0)	43 (16.2)	17 (6.4)	10 (3.8)	13 (4.9)
Virtual reality	186 (70.2)	1.67 (1.12)	79 (29.8)	117 (44.2)	42 (15.8)	8 (3.0)	9 (3.4)	10 (3.8)
Video games	186 (70.2)	1.55 (1.08)	79 (29.8)	134 (50.6)	27 (10.2)	10 (3.8)	5 (1.9)	10 (3.8)

^a^ICT: information and communication technology.

### Advantages and Disadvantages of ICT Tools for Fibromyalgia Management

Participants selected, on average, 1.99 (SD 1.31) advantages and 1.94 (SD 1.69) disadvantages. The most frequently selected perceived advantages of ICTs for pain management were the possibility of receiving treatment from home (n=156, 58.9%), access to specialized treatment (n=152, 57.4%), and use as a complement to face-to-face interventions (n=125, 47.2%). Conversely, the most frequent disadvantages were not having close contact with health professionals (n=132, 49.8%), not being able to express emotions and feelings (n=110, 41.5%), and a lack of knowledge or resources to use ICTs (n=80, 30.2%). See Table 4 for the complete list of advantages and disadvantages.

**Table 4:** Advantages and disadvantages of ICT^a^ tools.

Advantages and disadvantages			Participants (N=265), n (%)
**Advantages**			
	Possibility of receiving treatment from home	156 (58.9)	
	Access to specialized treatment	152 (57.4)	
	Use as a complement to face-to-face interventions	125 (47.2)	
	Reduction in economic cost	74 (27.9)	
	Anonymity	20 (7.5)	
**Disadvantages**			
	Not having close contact with the health professional	132 (49.8)	
	Not being able to express emotions and feelings	110 (41.5)	
	Lack of knowledge or resources to use ICT tools	80 (30.2)	
	Health professional not able to understand the patient’s nonverbal language	62 (23.4)	
	I would not be able to understand the health professional’s nonverbal language.	44 (16.6)	
	I would be concerned about data confidentiality.	27 (10.2)	
	There is less evidence about the use of ICTs for fibromyalgia management.	25 (9.4)	
	I would have not enough network bandwidth, or the connection could be broken.	24 (9.1)	
	Lack of legal regulations	13 (4.9)	

^a^ICT: information and communication technology.

### Relationships Between Study Variables

Regarding sociodemographic variables, older age was significantly correlated with a higher number of ICT tools used, a lower number of ICT-related advantages chosen, and lower satisfaction with many of the ICT tools listed (see Table 5). Higher levels of education were significantly correlated with a lower number of ICT tools used. However, higher education was significantly correlated with a greater number of advantages, fewer disadvantages, and a higher degree of satisfaction in relation to all the ICT tools listed (see Table 5).

Concerning pain-related variables, there was a significant correlation between fear of movement/injury (as assessed with the TSK-11) and a higher number of ICT tools used, as well as a greater number of disadvantages. There was also a significant negative correlation between TSK-11 scores and satisfaction with several ICT tools. Finally, FIQ-R scores were only significantly and positively related with the number of ICT tools used, along with scores from the CPGS Characteristic Pain Intensity Index, with lower satisfaction in the use of ICTs for tracking activity (see Table 5).

Results of the paired *t* tests conducted to compare the number of ICT tools used and the number of disadvantages reported were not significant when comparing between men and women and when comparing employment status (this variable was dichotomized in “unemployed” and “employed”). However, men pointed out significantly more advantages than women who participated in the survey (see Table 6). Additionally, when observing differences between people living alone versus people living with someone, it was found that those living alone significantly perceived more disadvantages than those living with someone. In addition, those who were not taking care of others perceived significantly more disadvantages than those who were.

**Table 5:** Correlations between sociodemographic and pain-related variables with the number of ICT^a^ tools used, advantages/disadvantages, and satisfaction with different types of ICTs.

Variables	Age	Education	Time since diagnosis	TSK-11^b^	FIQ-R^c^	CPGS^d^	
						Characteristic pain intensity	Disability score
Number of ICT tools used	0.18^f^	–0.23^e^	0.008	0.18^f^	0.14^f^	0.06	0.04
Number of ICT advantages	–0.22^e^	0.19^f^	–0.01	–0.02	–0.07	–0.08	–0.05
Number of ICT disadvantages	0.01	–0.12^f^	–0.05	0.21^e^	0.10	0.07	0.07
Satisfaction with fibromyalgia management websites	–0.12	0.19^f^	0.02	–0.16^f^	–0.09	–0.06	–0.03
Satisfaction with online multimedia resources	–0.15^f^	0.19^f^	–0.02	–0.17^f^	–0.10	–0.09	–0.03
Satisfaction with email	–0.19^f^	0.10	0.07	–0.13	–0.01	–0.02	0.02
Satisfaction with instant messaging apps	–0.24^e^	0.22^e^	–0.04	–0.15^f^	–0.1	–0.06	–0.04
Satisfaction with social networks	–0.23^e^	0.15^f^	–0.005	–0.08	–0.002	–0.09	0.02
Satisfaction with video conferences with health professionals	–0.25^e^	0.32^e^	–0.02	–0.21^f^	–0.13	0.001	0.04
Satisfaction with mobile phone apps	–0.03	0.09	0.02	–0.009	0.01	0.06	0.07
Satisfaction with phone calls with health professionals	–0.22^e^	0.13^f^	0.05	–0.15^f^	–0.01	0.03	0.09
Satisfaction with virtual reality	–0.12	0.12	–0.03	–0.004	–0.04	–0.007	0.01
Satisfaction with video games	–0.15^f^	0.20^f^	0.04	–0.13	–0.05	–0.08	–0.02
Satisfaction with technologies for tracking activity	–0.19^f^	0.22^e^	0.04	–0.24^e^	–0.13	–0.15^f^	–0.05
Satisfaction with digital sensors	–0.20^f^	0.19^f^	0.04	–0.22^f^	–0.07	–0.12	–0.04
Satisfaction with cameras to assess posture and movement	–0.17^f^	0.10	0.02	–0.09	–0.1	–0.03	–0.04
Satisfaction with platforms for following symptoms or schedules	–0.17	0.10	0.10	0.001	0.04	–0.05	0.02

^a^ICT: information and communication technology.

^b^TSK-11: 11-item Tampa Scale for Kinesiophobia.

^c^FIQ-R: Fibromyalgia Impact Questionnaire-Revised.

^d^CPGS: Chronic Pain Grade Scale.

^e^*P*<.001.

^f^*P*<.05.

**Table 6:** Paired *t* test comparisons.

Variables		Number of ICT^a^ tools used		Advantages		Disadvantages		
**Gender**								
	Women, mean (SD)	10.92 (4.46)	1.94 (1.30)		1.94 (1.71)			
	Men, mean (SD)	11.00 (4.87)	2.57 (1.33)		1.90 (1.48)			
	*t* Test (*df*)	–0.076 (262)	–2.124 (262)		0.098 (262)			
	*P* value	.939	**.**035^b^		.922			
**Employment status**								
	Unemployed, mean (SD)	11.35 (4.21)	1.93 (1.35)		2.10 (1.80)			
	Employed, mean (SD)	10.36 (4.80)	2.08 (1.24)		1.72 (1.49)			
	*t* Test (*df*)	1.774 (263)	–0.937 (263)		1.815 (263)			
	*P* value	.077	.350		.071			
**Living alone**								
	Yes, mean (SD)	11.56 (4.36)	1.90 (1.32)		2.80 (2.05)			
	No, mean (SD)	10.83 (4.50)	2.01 (1.31)		1.78 (1.57)			
	*t* Test (*df*)	0.965 (263)	–0.478 (263)		3.655 (263)			
	*P* value	.335	.633		**.**000^b^			
**Taking care of others**								
	Yes (mean; SD)	10.67 (4.43)	2.02 (1.25)		1.71 (1.66)			
	No (mean; SD)	11.19 (4.54)	1.97 (1.37)		2.15 (1.69)			
	*t* Test (*df*)	0.942 (263)	–0.278 (263)		2.153 (263)			
	*P* value	.347	.782		.032^b^			

^a^ICT: information and communication technology.

^b^Significant *P* values.

## Discussion

### Principal Findings

The study’s findings shed light on how individuals with fibromyalgia engage with a variety of ICT tools and their overall satisfaction regarding these resources. Only 0.75% of our participants reported never having used any of the tools listed, and the average number of ICT tools used per person was quite high (mean 10.94, SD 4.48, of 14 tools presented, although there was a high level of dispersion). Although we asked for the tools’ use for pain management, the participants probably answered thinking about the use they made of the tools for health in general; additionally, we do not know the use they attributed to each tool. For example, they may have tried a specific tool only once. However, in any case, it seems that they were open to using ICTs and trying different alternatives. Among the most used tools were instant messaging apps (89.4%), fibromyalgia management websites (88.3%). phone calls with health professionals (86.0%), and online multimedia resources (83.8%).

In addition to their willingness to use different ICT tools, satisfaction rates were low for each tool, as well as the average satisfaction index (mean 2.09, SD 0.38). This suggests that there is still room for improvement in the design of ICT tools for pain management, which is in agreement with prior reports [12]. More specifically, tools that participants were most satisfied with were instant messaging apps (mean 2.69, SD 1.41), phone calls with health professionals (mean 2.58, SD 1.45), fibromyalgia management websites aimed (mean 2.53, SD 1.30), and online multimedia resources (mean 2.40, SD 1.31). Participants chose an average of 1.99 advantages (SD 1.31) fibromyalgia management, with the most frequent ones being the possibility of receiving treatment from home (58.9%), access to specialized treatment (57.4%), and use as a complement to face-to-face interventions (47.2%). However, they also found a mean of 1.94 disadvantages (SD 1.69) being, with the most frequent ones being not having close contact with health professionals (49.8%), not being able to express emotions and feelings (41.5%), and a lack of knowledge or resources to use ICTs (30.2%).

Altogether, our results are related to the few available studies in the field with regard to several points. First, ICT tools used by most participants and with the greatest satisfaction in our study are directly related to the possibility of being able to communicate with a health care professional, highlighting the need for these patients to maintain close contact with health professionals. In this same line, as mentioned before, the third-most frequently chosen advantage of ICTs in our study was the possibility to receive remote treatments as a complement to face-to-face interventions; however, the 2 main perceived disadvantages of ICTs were related to losing contact with health professionals. These results are consistent with those of Cranen et al [15] since their participants especially valued the possibility of carrying out intermediate treatments, alternating in-person with remote treatment. The results also coincide with the findings of Cranen et al [14], who pointed out that patients with chronic pain appreciate that online interventions, although positively valued, should not be stand-alone treatments and should be complemented with face-to-face sessions.

Second, we found that among the most used ICT tools were fibromyalgia management websites and online multimedia resources. In the same line, Merolli et al [27] also made an important allusion to the fact that patients with chronic pain positively value those ICTs that allow them to look up information on the internet, in addition to being able to consult medical test results and receive personalized alerts and reminders.

Third, the most commonly highlighted advantage of ICTs in our study was being able to receive treatment remotely, from the comfort of one’s home, which is logical since people with fibromyalgia often have difficulty moving. This is also reflected in the work of Schneider and Hadjistavropoulos [13], who pointed out that ICTs are especially beneficial for those with mobility difficulties, which has also been highlighted as a classical advantage of using ICTs for health (see, for example, the classical work by Griffiths et al [28]).

Finally, among our participants, the third-most commonly cited disadvantage was the apprehension about inadequate ICT usage due to a lack of knowledge or resources. This echoes the findings of Schneider and Hadjistavropoulos [13], emphasizing that individuals demonstrating more interest and a favorable inclination toward ICTs are precisely those with higher perceived technology self-efficacy.

In our study, we found relationships among some of the studied variables that had not been previously reported in the existing literature. Primarily, older participants tended to have used more ICT tools, although they also seemed to perceive fewer advantages and less satisfaction. This might indicate that older people have more difficulty selecting tools that better fit their needs, so they try other alternatives. In addition, a certain digital divide related to age may exist, since older participants may not be as familiar with the use of digital technologies as younger participants and may have some problems using them (due to a lack of knowledge) [29], which could translate into a greater tendency to reject the technologies (and being related with higher dissatisfaction) or be inclined toward more “traditional” methods.

The opposite occurred regarding educational level: a significant negative correlation was found between the level of education and the number of ICT tools used. This phenomenon may be attributed to the enhanced knowledge of individuals who have access to superior educational levels, possibly resulting in being more selective when choosing between the wide range of ICTs at their disposal. Additionally, it should be noted that a relevant correlation was also found between educational level and the number of advantages/disadvantages selected regarding the use of ICTs; the higher the educational level of the participants, the more advantages and fewer disadvantages mentioned. This could be because education could have made it easier for these people to learn to better use technological devices and to know how to make better use of ICTs, in general terms, which would be reflected in their general opinion of ITCs and encourage them to adopt a favorable perspective regarding them. This previous idea would also be reinforced by the significant positive correlation that was found between the level of education and the degree of satisfaction with all the ICT tools listed in our study; by making better use of ICTs, overall satisfaction may have also improved for these participants.

Men reported significantly more advantages than women. This probably indicates a higher predisposition to use ICT tools for pain management. In contrast, results of Schneider and Hadjistavropoulos [13] revealed a higher level of interest in ICTs among female participants. Further investigation is needed regarding these findings because our sample was predominantly formed by women, and this result is difficult to interpret without prior studies having reported conclusive data on this subject. Finally, those living alone and those who were not taking care of others perceived significantly more disadvantages. The reasons for these differences merit further research since they are difficult to interpret.

Concerning pain-related variables, a negative correlation was found between the results of the TSK-11 and the mean satisfaction with the different ICT tools; the higher the degree of a general fear of movement/injury, the lower the satisfaction expressed regarding the use of ICT tools. This could be possibly explained by the general avoidance among people who present higher rates of kinesiophobia and fear avoidance, including their own exposure to treatments, whether in person or through ICTs.

### Limitations

As in other studies in our area, we also relied on quantitative cross-sectional data, making it difficult to obtain explanations. Qualitative studies could complement these findings by giving voice to participants to explain more in depth their perspectives on such issues. These studies could help provide a more comprehensive understanding of the research problem and contribute to the development of more effective solutions from the users’ perspective.

Furthermore, since the survey was conducted only once and not longitudinally, the participants provided a brief account of their current perceptions. This does not provide insight into how their views changed over time as they gained more knowledge and experience with the ICT tools, and we cannot establish causal relationships.

Finally, due to the constraints of the survey, we did not ask participants about how much they used each of the ICTs. Future research should consider this and study whether the perceptions change depending on the extent to which each technology is used.

### Conclusion

People with fibromyalgia are in favor of using tools that enable them to communicate with health care professionals. They also positively value those tools that grant them access to specialized online resources aimed at the management of their pain and general symptomatology. Moreover, remote treatment has been found to be particularly beneficial for those with mobility issues, which affect many people with fibromyalgia, as it allows them to receive care without having to leave their homes. However, important concerns about the ability to use ICTs due to a lack of knowledge or resources were observed. The age-related digital divide and education level have also been found to influence ICT tool usage and satisfaction. In addition, a negative association between satisfaction with ICTs and fear of movement/injury levels has been demonstrated, which may be attributed to a general tendency to avoid situations that elicit fear, including exposure to treatments that may be conceived as potentially painful, as long as therapeutic exercise patterns, while often effective, can be challenging for individuals with chronic pain, as they require significant effort and may momentarily exacerbate pain symptoms.

Results also indicate that despite the participants’ extensive experience with a large number of ICTs on average, both the satisfaction rates for each ICT tool listed in our study and the average satisfaction index were low, suggesting that there is ample scope for improvement in the design of ICT tools aimed at pain management, as well as formulate effective strategies aimed at reinforcing perceived self-efficacy of users.

## Abbreviations

CPGS: Chronic Pain Grade Scale
FIQ-R: Fibromyalgia Impact Questionnaire-Revised
ICBT: internet-based cognitive behavioral therapy
ICT: information and communication technology
TSK-11: 11-item Tampa Scale for Kinesiophobia

## Data Availability

The data sets generated and analyzed during this study are available from the corresponding author upon reasonable request.
